# Washed microbiota transplantation for ribotype 027 *Clostridioides difficile* infection in a pregnant woman with a two-year follow-up: A case report

**DOI:** 10.7555/JBR.39.20250063

**Published:** 2025-05-27

**Authors:** Xinyi He, Sibusiso Luthuli, Quan Wen, Chuan Wang, Jinli Ding, Bota Cui, Faming Zhang

**Affiliations:** 1 Department of Microbiota Medicine & Medical Center for Digestive Diseases, the Second Affiliated Hospital of Nanjing Medical University, Nanjing, Jiangsu 210011, China; 2 Key Lab of Holistic Integrative Enterology, the Second Affiliated Hospital of Nanjing Medical University, Nanjing, Jiangsu 210011, China; 3 General Medical Unit, Steve Biko Academic Hospital, University of Pretoria, Pretoria 0002, South Africa; 4 Department of Gastroenterology, the Affiliated Jiangning Hospital of Nanjing Medical University, Nanjing, Jiangsu 211100, China

**Keywords:** fecal microbiota transplant, pregnant women, *Clostridioides difficile*

## Abstract

*Clostridioides difficile* (*C. difficile*) is one of the major causes of nosocomial infections. Pregnant women, who are generally considered at low risk for *C. difficile* infection (CDI), have attracted attention because of an increasing number of reports. Oral vancomycin, the only first-line treatment for pregnant women infected with *C. difficile*, has been associated with increasing strain resistance, leading to decreased efficacy. Fecal microbiota transplantation (FMT) is recommended for severe, fulminant, and recurrent CDI; however, it is generally avoided in pregnant women because of safety concerns. We report a case of a pregnant woman with a primary ribotype 027 CDI who experienced a successful outcome with washed microbiota transplantation (WMT), an improved form of FMT, *via* enema. The specific strain of ribotype 027 is related to severe outcomes but has not previously been reported in pregnant women. The follow-up lasted for two years, during which the patient's diarrhea was fully alleviated without recurrence. The baby showed normal growth and development, and no adverse events were recorded for either. This case provides evidence for the efficacy and safety of WMT in pregnant women infected with *C. difficile*, indicating that WMT *via* enema may be a viable therapeutic strategy for this population for treating CDI.

**Introduction**

Oral vancomycin is currently the only first-line treatment for pregnant women infected with *Clostridioides difficile* (*C. difficile*). Nevertheless, *C. difficile* has shown reduced susceptibility to vancomycin^[1]^. Fecal microbiota transplantation (FMT), which refers to the transplantation of fecal microbiota from a healthy donor to a recipient's gastrointestinal tract, is recommended for severe, fulminant, and recurrent *C. difficile* infection (rCDI); however, it is not recommended for pregnant women because of safety concerns^[2]^.

Ribotype 027 is an independent predictor of severe CDI and mortality^[3]^, and it has rarely been reported in China, with no previous reports involving pregnant women. Here, we present the first case of successful application of washed microbiota transplantation (WMT) *via* enema for the treatment of primary ribotype 027 CDI in a pregnant woman. Ethical approval (2019-KY-091) was granted by the Ethics Committee of the Second Affiliated Hospital of Nanjing Medical University (Nanjing, Jiangsu, China). Informed consent was obtained from the patient.

Different FMT protocols influence efficacy and safety in the treatment of CDI^[4–5]^. The novel method of FMT based on an automatic washing process, referred to as WMT^[5]^, was introduced in a 2019 consensus statement^[6]^. Compared with the manual method, the automatically washed preparation significantly reduced FMT-related adverse events (AEs)^[5]^. Meanwhile, 90.7% (49/54) of patients infected with *C. difficile* achieved clinical cure after WMT^[7]^. In this case, the successful outcome over the two-year follow-up support further research to determine whether WMT *via* enema could become a standard treatment for pregnant women infected with *C. difficile*.

## Case presentation

A 26-year-old woman at nine weeks and five days of gestation reported diarrhea lasting over one month and was admitted to the authors' affiliated hospital on December 8, 2022. The diarrhea, occurring approximately six times per day, started on October 26, 2022. The patient took a single cefdinir capsule, which significantly improved her symptoms. However, after the medication was discontinued, diarrhea with mucus and bloody stools recurred, occurring four times per day.

**Figure 1:**
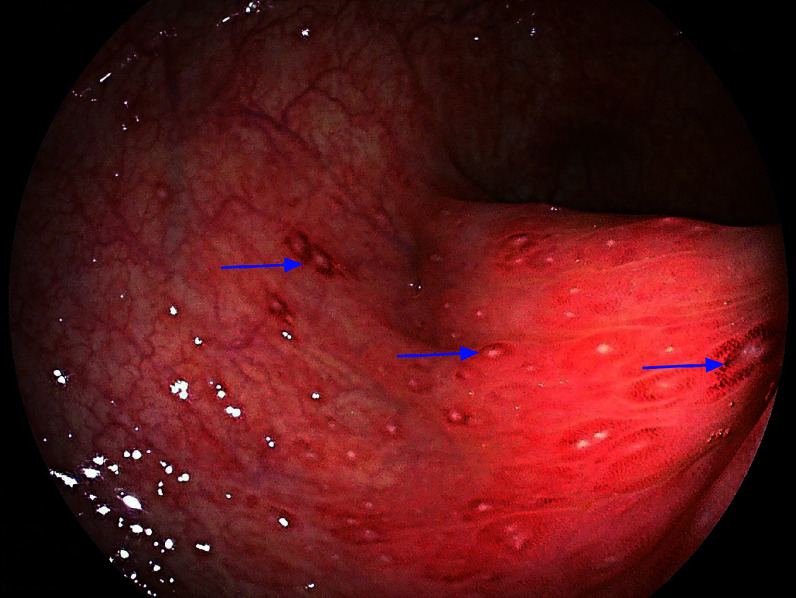
Colonoscopy of the sigmoid colon. Colonoscopy revealed multiple punctate erosions and flaky ulcers covered with white exudate (arrows) in the sigmoid colon.

The patient was initially admitted to the hospital on November 5, 2022, at which time it was discovered that she was five weeks pregnant. Routine stool examination revealed 3+ leucocytes and 1+ erythrocytes per high-power field, along with positive occult blood. No abnormalities were found in the complete blood routine, fecal bacterial culture, or rotavirus testing. The patient was discharged after nine days of intravenous and oral cephalosporin antibiotic treatment, with no improvement in symptoms. A colonoscopy without anesthesia was performed on November 21, 2022. It was advanced only the junction of the sigmoid colon and the descending colon due to safety concerns, showing diffuse punctate lesions and flaky ulcers covered with white moss in the sigmoid colon (***Fig. 1***). Two sigmoid biopsies and one rectal biopsy showed active chronic enteritis, accompanied by erosion and interstitial congestion.

The patient was admitted to our hospital on December 8, 2022, with presentations similar to those during her previous admission. A medication history revealed oral doxycycline use (200 mg/day) for 22 days in July 2022 for acne treatment. No abnormalities were detected on physical examination. The PCR assay (Xpert® *C. difficile*/Epi, Cepheid, Sunnyvale, CA) identified the ribotype 027 *C. difficile* by detecting the presence of the toxin B gene, the binary toxin gene, and the deletion at position 117 of the *tcdC* gene. A routine stool examination revealed positive occult blood. Complete blood count, liver and kidney function tests, and fecal bacterial culture were normal. Based on the clinical symptoms, colonoscopy, and PCR results, the diagnosis was ribotype 027 CDI.

We chose WMT as the therapy, with informed consent from the patient. No antibiotics were used. Fresh allogeneic microbiota from one donor unit was delivered by enema three times over three consecutive days. We defined 10 cm^3^ of microbiota precipitate as one unit in the China Microbiota Transplantation System (CMTS)^[5]^. The diarrhea disappeared after the second infusion, and the same PCR test for *C. difficile* showed a negative result one month after WMT. The patient did not undergo another colonoscopy because of the absence of diarrhea, which was considered a clinical cure. On July 4, 2023, the patient gave birth *via* cesarean section. The follow-up lasted for two years; no diarrhea was reported, and the baby exhibited normal growth and development. Meanwhile, no AEs were recorded for either the patient or the baby.

## Discussion

CDI in pregnant women has attracted attention because of the increasing prevalence of *C. difficile* and the limited treatment options available for this population. In our case, we faced the challenge of managing primary CDI. The pregnant woman infected with ribotype 027 *C. difficile* achieved clinical cure with WMT. The diarrhea resolved, and the infant exhibited normal growth and development during the two-year follow-up.

When WMT was used as a treatment option, the clinical cure rates were comparable between patients with primary CDI and recurrent CDI (91.89% *vs.* 88.23%, *P* = 0.645), with more than half of the patients with primary CDI undergoing WMT alone, highlighting its therapeutic potential^[7]^.

The presence of ribotype 027 *C. difficile*, a strain associated with severe outcomes but not previously reported in pregnant women, posed a challenge^[3]^. Recent data demonstrated that ribotype 027 accounted for the highest proportion (77.4% [41/53]) in cases of reduced vancomycin susceptibility, which was associated with declining initial cure rates and sustained clinical response^[1]^. Fortunately, FMT achieved a rate of 89% (32/36) for symptoms caused by ribotype 027 rCDI, with four non-responders having serious pre-existing conditions^[8]^. These factors prompted us to choose FMT alone as the strategy.

FMT is limited in pregnant women because of safety concerns, including the risk of microbiota-related adverse events (MRAEs) and delivery-related adverse events (DRAEs). As an improved method of FMT, WMT was designed based on an automatic washing process and related delivery considerations^[5]^. Compared with the traditional methods, WMT-related adverse event rates were significantly lower. The intraperitoneal injection model demonstrated the important role of washed fecal microbiota preparations in mice, which may be further explained by decreased inflammatory substances such as leukotriene B4, prostaglandin G2, and others^[5]^. Additionally, a strict donor screening mechanism, under which the donor qualification rate was as low as 3.1% (32/1036), reduced the risk of MRAEs from the source^[6]^. According to statistics from 2000 to 2020, AEs were more common in patients who underwent FMT *via* upper gastrointestinal (GI) routes than in those *via* lower GI routes (28.8% *vs.* 17.5%)^[9]^. Upper GI routes included capsule, mid-gut tube, and gastroscopy, while lower GI routes included colonoscopy, colonic transendoscopic enteral tube (TET), and enema. The invasive operation involved in colonoscopy and colonic TET could be a threat to the pregnant woman; therefore, we selected enema administration to reduce the risk of DRAEs.

While ensuring safety, efficacy must also be considered. A systematic review illustrated that FMT protocols, including administration routes and the number of infusions, influence efficacy in treating rCDI^[4]^. FMT *via* lower GI is superior to that *via* upper GI for treating recurrent and refractory CDI (95% *vs.* 88%, *P* = 0.02)^[10]^. Although enema was associated with lower efficacy rates after a single infusion, multiple infusions significantly improved treatment outcomes^[4]^. In our case, the disease was not severe or complicated, and WMT was administered *via* enema once daily for three consecutive days.

The main limitation of our report is that no firm conclusion can be drawn from a single case. Future research is needed to provide stronger evidence that WMT *via* enema may be a viable strategy for pregnant women infected with CDI. In clinical practice, the treatment strategy should be selected based on disease severity and patient-specific conditions.
